# A Rare Cause of Nasal Obstruction: Metastatic Renal Cell Carcinoma

**DOI:** 10.1155/2016/2560749

**Published:** 2016-11-09

**Authors:** Tuba Dilay Kokenek-Unal, B. Gumuskaya, B. Ocal, Murat Alper

**Affiliations:** ^1^Kayseri Research and Training Hospital, Department of Pathology, Kayseri, Turkey; ^2^Yildirim Beyazit University, Department of Pathology, Ankara, Turkey; ^3^Diskapi Yildirim Beyazit Research and Training Hospital, Department of Ear-Nose-Throat, Ankara, Turkey; ^4^Diskapi Yildirim Beyazit Research and Training Hospital, Department of Pathology, Ankara, Turkey

## Abstract

*Introduction*. Renal cell carcinoma can present with several interesting symptoms, paraneoplastic syndromes, and unusual metastatic sites. Head and neck region is one of the rare locations for renal cell carcinoma metastasis.* Case Report*. A 50-year-old man was admitted to the hospital with nasal congestion and snoring. Physical examination revealed nasal serous secretion. First taken biopsy was misinterpreted. The symptoms of the patient were not revealed and he was readmitted to the hospital. On radiologic examination, a vascular rich mass in maxillary sinus extending to the nasal cavity was observed. Biopsy was diagnosed as renal cell carcinoma metastasis. Herein, we present a patient with renal cell carcinoma presenting nasal obstruction and snoring as first and recurrent symptom.

## 1. Introduction

Malignant tumors of nasal cavity and paranasal sinuses account for less than 1% of all malignant cancers [1]. Most of carcinoma seen in this region is primary tumor and metastatic lesions are rarely seen. Following the breast and lung carcinoma, renal cell carcinoma is the third most common cancer which metastasizes to the head and neck [2]. Renal cell carcinoma has tendency to atypical metastasis and it can be initial presentation [3]. Head and neck metastasis is observed in 15% of patients with renal cell carcinoma [4]. Metastatic renal cell carcinoma to the paranasal sinuses represents epistaxis (70%), nasal obstruction, facial pain, or orbital mass [5].

## 2. Case Report

**Figure 1:**
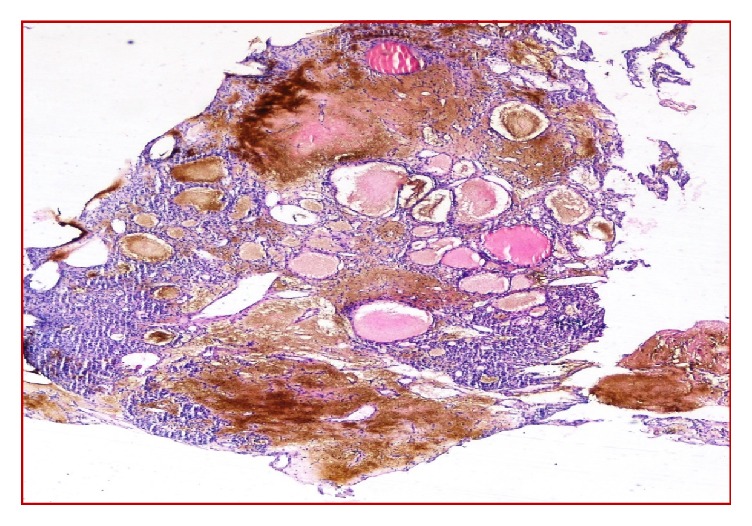
Dilated blood vessels in a loose stroma (H&E, ×10).

**Figure 2:**
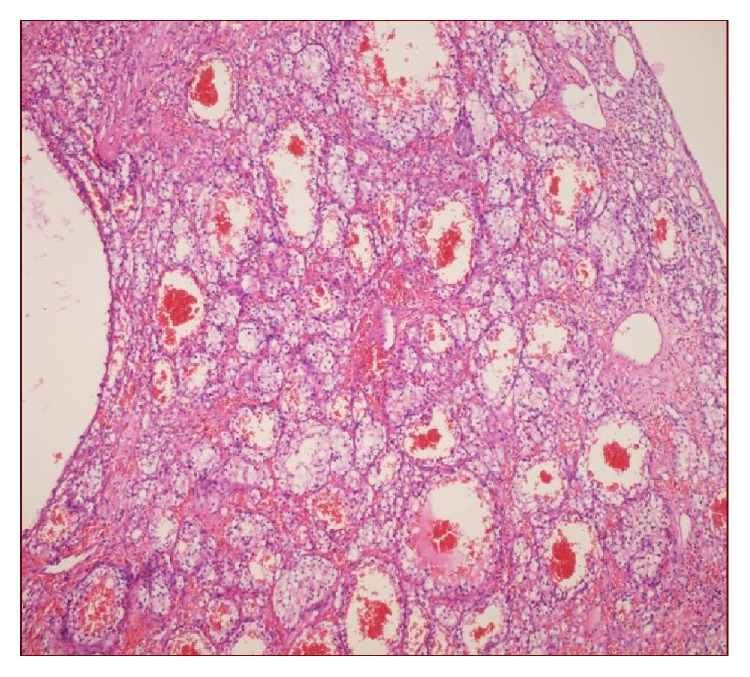
Dilated blood vessels and cells clusters with clear cytoplasm in congested loose stroma (H&E, ×20).

**Figure 3:**
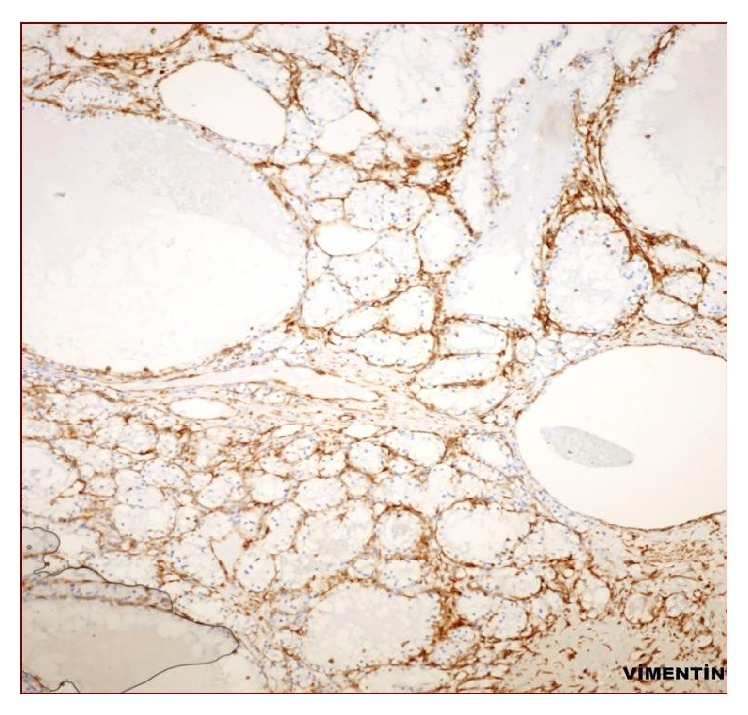
Clear cells, positively stained by Vimentin (×40).

**Figure 4:**
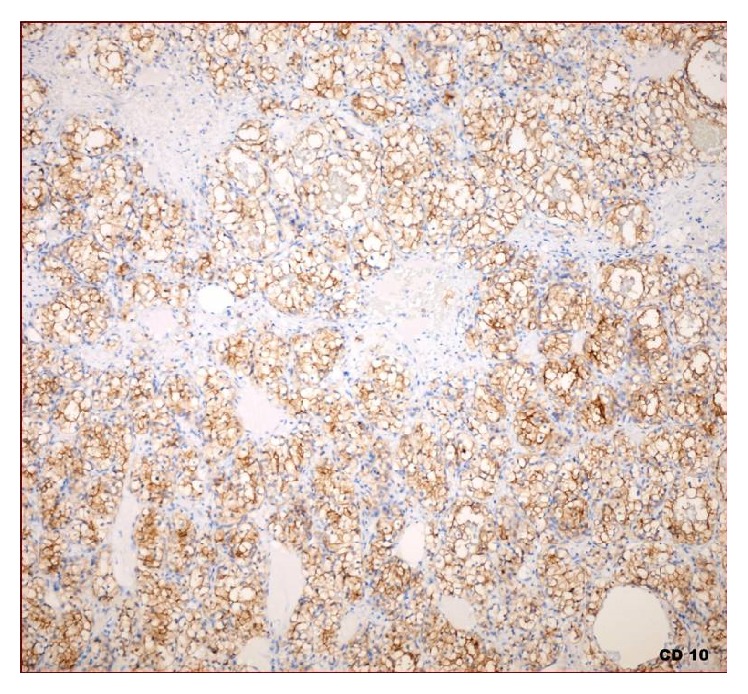
Clear cells, positively stained by CD10 (×20).

A 50-year-old man has been suffering from nasal obstruction and snoring for 2 months and was admitted to our hospital. His medical history revealed that he had total thyroidectomy and an operation for nasal septum deviation 30 years ago. He did not complain about nasal discharge, nasal itching, or sneezing. On physical examination, there was a serous secretion on nasal mucosa and nasal septum deviated to the right in the posterior portion. A small biopsy was taken and sent to the pathology. Brown-black coloured and soft specimen was measured 0.7 × 0.4 × 0.2 cm in size. In microscopic evaluation, several dilated blood vessels in a loose stroma were observed (Figure 1).  A few cells with foamy cytoplasm were seen and were interpreted as histiocytes and they were reported as vascular rich lesion suggesting hemangioma. A few months later, the patient was readmitted to the hospital with similar complaints and additionally with a swelling of left half of his face. On sinonasal MRA, a heterogeneous mass was seen in maxillary sinus, extending to the nasal cavity and infiltrating nasal conchae. The patient underwent an operation. The maxillary sinus was curetted, and several biopsies were taken from sinus mucosa. On histologic examination, largely dilated, thin-walled vessels were observed in a loose stroma similar to the first biopsy. However, between blood vessels, few histiocyte-like cells with bland nucleus and foamy/clear cytoplasm stood out without forming a distinctive pattern (Figure 2). Although the patient does not have any complaint of flank pain or hematuria, metastatic renal cell carcinoma was suspected and urgent urologic consult was asked for. CD10, EMA, PanCK, and Vimentin immunohistochemistry were applied to determine the origin of the clear cells. These cells were stained positively with all these antibodies. (See Figures 3 and 4; for Vimentin and CD10 staining, resp.) In addition to that, on abdominal CT, a heterogeneous exophytic mass was observed. It was 12 × 12 × 10 cm in size, located in middle-to-upper portion of left kidney, and had cystic and necrotic areas. The sinonasal biopsy was diagnosed as metastatic renal cell carcinoma. Surgical and medical treatment of the patient was made in another hospital. One year later, the lesion recurred and the patient came up again in ear-nose-throat clinic with similar signs and symptoms. On physical examination, there was a polypoid mass extending to inferior nasal concha of left nasal cavity. Total maxillectomy was made and specimen was reported again as metastatic renal cell carcinoma. Any solid organ metastasis was not detected in this time.

## 3. Discussion

Renal cell carcinoma is third common cancer leading death in the world [6]. Clinical manifestation of renal cell carcinoma is various and classic symptoms such as flank pain and palpable mass are seen only in 10% of cases [2, 3]. Because of slow growth of renal cell carcinoma in initial stages and propensity to metastasize even in small sizes, approximately 25% of cases have distant metastasis at initial diagnosis [3, 4]. In 15% of cases, renal cell carcinoma metastasizes to head and neck region; only 1% of patients do not have any other metastasis [7]. There are several theories proposed to explain affinity of neoplastic cells for sinonasal region: arterial microemboli, Batson's venous plexus, and lymphatic way [7]. As in our case, histopathological evaluation can be challenging in especially small and necrotic biopsies because both sinonasal region and metastatic foci of renal cell carcinoma are highly vascular tissues. Clinical and radiological findings are very helpful to leading correct diagnosis. In our case, renal cell carcinoma was presented initially with paranasal sinus metastasis and it recurred also in the same region one year after radical nephrectomy while taking immunotherapy. After nephrectomy, metastasis can be observed in approximately 30% of cases [8]. Prognosis of metastatic renal cell carcinoma is generally poor with a survival of less than one year [4]. Moreover, patients with atypical metastasis have approximately similar prognosis and survival rates with typical metastasis such as lung metastasis [9]. Metastasectomy is the best treatment for atypical metastasis for renal cell carcinoma [9]. In conclusion, renal cell carcinoma is very interesting tumor with atypical metastasis and should be kept in mind in differential diagnosis of nasal obstruction and epistaxis. In addition to that long term follow-up is strongly recommended for the patients with renal cell carcinoma.
